# Prätherapeutische Dysphagie bei Kopf-Hals-Tumor-Patienten

**DOI:** 10.1007/s00106-021-01128-8

**Published:** 2022-02-04

**Authors:** Uta Lehner, Eugen Zaretsky, Almut Goeze, Laura Wermter, Boris A. Stuck, Richard Birk, Andreas Neff, Ingo Fischer, Shahram Ghanaati, Robert Sader, Christiane Hey

**Affiliations:** 1 Abteilung für Phoniatrie und Pädaudiologie, HNO, Universitätsklinikum Gießen und Marburg GmbH, Standort Marburg, Philipps-Universität Marburg, Baldingerstr. 1, 35043 Marburg, Deutschland; 2 Klinik für Hals‑, Nasen- und Ohrenheilkunde, Universitätsklinikum Gießen und Marburg GmbH, Standort Marburg, Philipps-Universität Marburg, Marburg, Deutschland; 3 Klinik und Poliklinik für Mund‑, Kiefer- und Gesichtschirurgie, Universitätsklinikum Gießen und Marburg GmbH, Standort Marburg, Philipps-Universität Marburg, Marburg, Deutschland; 4 Klinik für Mund‑, Kiefer‑, Plastische Gesichtschirurgie, Universitätsklinikum Frankfurt/Main, Goethe-Universität Frankfurt/Main, Frankfurt/Main, Deutschland

**Keywords:** Schluckstörung, Body-Mass-Index, EAT-10, Aspiration, Malnutrition, Deglutition disorders, Body mass index, EAT-10, Aspiration, Malnutrition

## Abstract

**Hintergrund:**

Sowohl der Schluck- als auch der Ernährungsstatus bei Kopf-Hals-Tumor(KHT)-Patienten nach einer onkologischen Therapie sind gut untersucht. Prätherapeutisch werden sie aber selten thematisiert, obwohl diese den Erfolg einer onkologischen Therapie nachhaltig beeinflussen können.

**Ziel der Arbeit:**

Ziel dieser Arbeit ist die systematische Erfassung des Schluck- und Nutritionsstatus von KHT-Patienten vor Beginn einer onkologischen Therapie.

**Material und Methoden:**

Bei 102 Patienten wurden zur objektiven Erfassung des Schluckvermögens endoskopisch die Penetration/Aspiration via PA-Skala (PAS), die Oralisierungseinschränkung (Functional Oral Intake Scale, FOIS) und die Versorgungsrelevanz (VRS) erhoben. Die subjektive Einschätzung des Schluckvermögens erfolgte via Fragebogen gEAT-10 („German EAT-10“), die orientierende Erfassung des Nutritionsstatus via Body-Mass-Index (BMI). Schluckvermögen und BMI wurden uni- und multivariat auf mögliche Einflussfaktoren geprüft.

**Ergebnisse:**

Auffällige PAS-, FOIS- und VRS-Werte wurden bei ≤ 15 % der Patienten festgestellt. Der BMI war häufiger zu hoch als zu niedrig. Das objektiv erfasste Schluckvermögen war v. a. vom Tumorstadium abhängig und korrelierte mittelstark mit gEAT-10. Der gEAT-10-Gesamtscore war auffällig. Der Nutritionsstatus war von Patientengeschlecht und VRS abhängig.

**Schlussfolgerung:**

Prätherapeutisch zeigte sich bei der Mehrzahl der Patienten keine Dysphagie oder Malnutrition. Ein auffälliges Schluckvermögen war mit höheren Tumorstadien assoziiert, eine Malnutrition mit weiblichem Geschlecht und Versorgungsrelevanz. Nichtsdestotrotz sollte hinsichtlich moderner onkologischer Therapie der Schluck- und Nutritionsstatus bei KHT-Patienten bereits prätherapeutisch systematisch erfasst werden, um ein optimales Patienten-Outcome zu erzielen.

## Abstract (en)

**Background:**

The swallowing and nutritional status of head-and-neck cancer patients after oncological therapy have been extensively researched. However, the same topics are seldom scrutinized before the onset of oncological therapy, although they can influence treatment success in the long term.

**Objective:**

This study focusses on a systematic assessment of swallowing function and nutritional status in head-and-neck cancer patients prior to oncological therapy.

**Materials and methods:**

In 102 patients, penetration/aspiration (PA scale), limitations of oral intake (Functional Oral Intake Scale, FOIS), and the need for further intervention (NFI) were endoscopically assessed to objectively quantify swallowing function. The subjective evaluation of swallowing function was carried out with the gEAT-10 (German EAT-10) questionnaire, nutritional status was assessed by body mass index (BMI). Possible impact factors for swallowing function and BMI were analyzed by univariate and multivariate methods.

**Results:**

PAS, FOIS, and NFI values were abnormal in ≤ 15% of patients. BMI was more often too high than too low. Objectively assessed swallowing functions depended predominantly on tumor stage and showed moderate correlations with gEAT-10. The latter mostly yielded a “fail” result. The nutritional status depended on the patients’ biological sex and NFI.

**Conclusion:**

In the pre-treatment setting, neither dysphagia nor malnutrition were found in most patients. Impaired swallowing was associated with higher tumor stages, malnutrition with female sex and NFI. A systematic pre-treatment assessment of swallowing and nutritional status in head-and-neck cancer patients appears necessary for modern oncological therapy and optimal patient outcome.

Die einschneidenden Funktionsstörungen infolge einer Kopf-Hals-Tumor-Erkrankung können die Lebensqualität und soziale Teilhabe der Betroffenen erheblich beeinträchtigen. Schluckstörungen führen darüber hinaus zu einer Steigerung von Morbidität und Mortalität und sind damit nicht nur für den Patienten, sondern auch von sozioökonomischem Interesse. Eine frühzeitige systematische objektive wie subjektive Erfassung einer möglichen Schluckstörung vor onkologischem Therapiebeginn könnte den onkologischen Krankheitsverlauf entscheidend beeinflussen und ist daher Gegenstand der vorliegenden Studie.

Schluckstörungen infolge einer onkologischen Therapie bei Kopf-Hals-Tumor-Patienten sind gut belegt [1–3], während die Ermittlung ihrer Prävalenz vor Beginn einer onkologischen Therapie weniger im wissenschaftlichen Fokus steht [4, 5]. Dabei bestehen Hinweise, dass mögliche Folgekomplikationen, wie v. a. die Malnutrition, Auswirkungen auf den Verlauf einer onkologischen Kopf-Hals-Tumor-Erkrankung nehmen. So identifizierten Tsai et al. [6] eine präoperative Malnutrition als unabhängigen Prädiktor für postoperative Komplikationen bei Kopf-Hals-Tumor-Patienten. Chang et al. [7] belegten einen signifikanten Zusammenhang zwischen niedrigem prätherapeutischem Body-Mass-Index (BMI) und frühem Versterben eines Patienten mit Kopf-Hals-Tumor noch während der onkologischen Behandlung. Dabei wird die Malnutrition einer onkologischen Erkrankung meist auf deren konsumierenden Charakter zurückgeführt [8], konnte jedoch von Jager-Wittenaar et al. [9] und Ottoson et al. [10] als relevante Folge einer Schluckstörung nachgewiesen werden.

Diese Studien legen die Notwendigkeit nahe, bereits prätherapeutisch den Dysphagiestatus eines Kopf-Hals-Tumor-Patienten systematisch und detailliert zu erfassen, um die peri- und posttherapeutische Versorgung von Schluckstörungen sicherzustellen und angepasst an den individuellen Bedarf besser planen zu können. Mithilfe der instrumentellen Diagnostik können dabei nicht nur die Penetration/Aspiration und Oralisierungseinschränkung, sondern auch die sich aus diesen beiden Kernproblemen ergebende Versorgungsrelevanz erhoben werden.

Dabei bezieht die moderne onkologische Versorgung den Patienten mit seinen Interessen aktiv in den Therapieprozess mit ein, sodass neben der objektiven Erhebung einer Schluckstörung auch die subjektive Einschätzung des Patienten in Bezug auf Umfang und Schwere der Dysphagie von Relevanz ist. Hierfür eignet sich der international anerkannte Fragebogen EAT-10 [11], der als gEAT-10 in deutscher für Kopf-Hals-Tumor-Patienten validierter Übersetzung vorliegt [12]. Er besteht aus insgesamt zehn 5‑Punkte-Likert-skalierten Fragen (0 „kein Problem“ und 4 „schwerwiegendes Problem“), die neben spezifischen Symptomen auch soziale und emotionale Aspekte einer Schluckstörung berücksichtigen.

Ziel der vorliegenden Arbeit war die systematische Untersuchung des Schluckvermögens bei Kopf-Hals-Tumor-Patienten, v. a. der Penetration/Aspiration, Oralisierungseinschränkung und Versorgungsrelevanz, vor Beginn einer onkologischen Therapie. Zusätzlich erfolgte die orientierende Erfassung des Ernährungsstatus mittels BMI. Zudem war von Interesse, inwieweit Tumorgröße, -lokalisation, Patientenalter und -geschlecht Einfluss auf die Schluckfähigkeit nehmen und inwiefern die objektive Erfassung der Schluckfunktion mit der subjektiven Patienteneinschätzung übereinstimmt.

## Methodik

Für diese Studie liegen Ethikvoten aus zwei Kliniken vor: Universitätsklinikum Frankfurt/Main (AZ 240/10) und Universitätsklinikum Marburg (AZ 63/15).

### Stichprobe

Die Durchführung der vorliegenden Studie erfolgte prospektiv im Zeitraum von August 2015 bis Mai 2021 in der Hals-Nasen-Ohrenheilkunde und Mund‑, Kiefer- und Gesichtschirurgie des Universitätsklinikums Marburg sowie in der Mund‑, Kiefer‑, Plastischen Gesichtschirurgie des Universitätsklinikums Frankfurt/Main.

Für die Studie wurden Patienten mit einem neu diagnostizierten Mundhöhlen‑, Oropharynx‑, Larynx- bzw. Hypopharynxtumor, Stadium I–IV (UICC, Union International Contre le Cancer), vor Beginn einer onkologischen Therapie rekrutiert. Inkludiert wurden Patienten im Alter ≥ 18 Jahre, mit schriftlicher Einwilligungserklärung und vollständigem Datensatz. Ausschlusskriterien bildeten das Vorliegen eines Kopf-Hals-Tumor-Rezidivs sowie weitere schluckbeeinträchtigende Erkrankungen wie z. B. Encephalitis disseminata.

**Figure 1:**
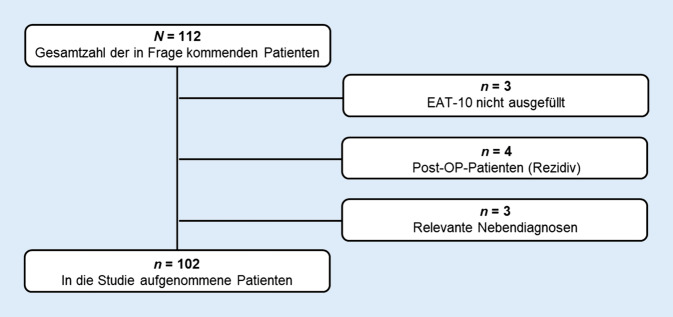

Für die Studie wurden 112 Patienten rekrutiert, wovon 10 aufgrund nicht zutreffender Ein- bzw. Ausschlusskriterien exkludiert wurden (Abb. 1).

Insgesamt wurden demnach 102 Patienten, 18 weiblich (17,6 %), 84 männlich (82,4 %), im Alter von im Mittel 62,6 ± 8,5 Jahren in die Studie aufgenommen (Tab. 1).

**Table 1:** 

Patientencharakteristika	Gesamtstichprobe
	*n* (%)
*Tumorstadium*	
I	5 (4,9)
II	13 (12,7)
III	22 (21,6)
IV	62 (60,8)
*Tumorlokalisation*	
Mundhöhle	19 (18,6)
Oropharynx	45 (44,1)
Larynx- bzw. Hypopharynx	38 (37,3)
*Alter (in Jahren)*	
≤ 40	0 (0,0)
41–60	41 (40,2)
> 60	61 (59,8)

Zwischen Männern und Frauen bestanden keine Altersunterschiede laut Mann-Whitney-U-Test (*p* > 0,05).

### Untersuchungsablauf

Alle Patienten erhielten eine FEES^®^-Diagnostik (flexible endoskopische Evaluation des Schluckvorgangs) nach Langmore-Protokoll zur objektiven Erfassung möglicher Penetration/Aspiration, Oralisierungseinschränkung und Versorgungsrelevanz einer Schluckstörung, inklusive der anatomisch-physiologischen Untersuchung, der Schluckfunktionsprüfung sowie der Überprüfung von Haltungspositionen und Schluckmanövern [13, 14]. Für die Schluckfunktionsprüfung wurden standardisiert flüssige und pürierte (in den Volumina 2, 5, 10 und 20 ml) sowie feste Konsistenzen eingesetzt, sofern das Schluckvermögen des Patienten dies erlaubte.

Die Schweregradeinteilung der Penetration/Aspiration erfolgte mit der Penetrations-Aspirations-Skala (PAS) nach Rosenbek ([15, 16]; Infobox 1), die der Oralisierungseinschränkung mit der Functional Oral Intake Scale (FOIS) nach Crary ([17]; Infobox 2). Die Originalreihenfolge der FOIS wurde für statistische Analysen invertiert und damit an die Reihenfolge der PAS adaptiert [18]. Eine schwerwiegende Penetration bestand bei PAS 4–5, Aspiration ab PAS ≥ 6, eine therapierelevante Oralisierungseinschränkung ab FOIS ≥ 4. Die Versorgungsrelevanz (VRS) einer Schluckstörung wurde auf Basis der ersten beiden Kardinalsymptome erschlossen und wie folgt festgelegt: PAS ≥ 4 bzw. FOIS ≥ 4.

Die Erfassung der subjektiven Wahrnehmung des Schluckvermögens aus Sicht der Patienten erfolgte über den Fragebogen gEAT-10 (German Eating Assessment Tool, Tab. 4).

### Statistische Analysen

Als Erstes erfolgte die deskriptiv statistische Aufbereitung des Auftretens von PAS, FOIS und VRS sowohl für die Gesamtstichprobe als auch für einzelne Tumorstadien, -lokalisationen, Altersgruppen und Geschlecht, des Weiteren die des BMI-Werts, Gewichtsverlusts (aktuelles Gewicht in Bezug auf Normalgewicht vor Erkrankungsbeginn) und des gEAT-10-Gesamtscores.

Der Zusammenhang zwischen PAS, FOIS, VRS sowie BMI und den möglichen Einflussfaktoren Tumorstadium, -lokalisation, Patientenalter und -geschlecht wurde univariat untersucht. Dies erfolgte mit nichtparametrischen Testverfahren aufgrund des ordinalen Skalenniveaus von PAS, FOIS und VRS.

Der Zusammenhang zwischen PAS, FOIS, VRS sowie BMI und UICC-Tumorstadien wurde mittels Spearman-Korrelationen berechnet.

Die Werteverteilung von PAS, FOIS, VRS und BMI wurde abhängig von der Tumorlokalisation zuerst mit Kruskal-Wallis-H-Tests geprüft. Die anschließenden paarweisen Vergleiche einzelner Tumorlokalisationen erfolgten mit Mann-Whitney-U-Tests, ebenso wie die Untersuchung der Werteverteilung abhängig vom Patientengeschlecht.

Die Zusammenhangsstärke zwischen Patientenalter sowie PAS, FOIS, VRS und BMI wurde via Spearman-Korrelationen geprüft.

Darüber hinaus wurde der BMI mit allen drei Parametern (PAS, FOIS, VRS) korreliert.

Die Analyse möglicher Einflussfaktoren auf PAS, FOIS und VRS erfolgte mit drei linearen Regressionen (Methode „Einschluss“), mit den unabhängigen Variablen Tumorstadium und -lokalisation sowie Patientengeschlecht und -alter. Die Aussagekraft des jeweiligen Gesamtmodells wurde mit einer ANOVA bestimmt und die erklärte Varianz mit dem korrigierten Nagelkerkes R^2^. Die Einflussstärke der einzelnen überprüften Faktoren wurde mit standardisierten Beta-Koeffizienten ermittelt.

In einer weiteren linearen Regression wurden BMI als abhängige, dagegen Tumorstadium, -lokalisation, Patientengeschlecht und -alter sowie VRS als unabhängige Variablen eingesetzt.

Die Zusammenhangsanalyse der objektivierbaren (PAS, FOIS und VRS) mit der subjektiv empfundenen Einschätzung des Schluckvermögens (gEAT-10-Gesamtscore und -Einzelitems) erfolgte via Spearman-Korrelationen. Für die erste gEAT-10-Frage (subjektiver Gewichtsverlust) wurde zusätzlich eine Spearman-Korrelation mit dem objektiv erfassten Gewichtsverlust berechnet.

## Ergebnisse

Von allen 102 Patienten wiesen 6 (5,9 %) eine schwerwiegende Penetration und 8 (7,8 %) eine Aspiration auf. Eine therapierelevante Oralisierungseinschränkung fand sich bei 7 (6,9 %), eine versorgungsrelevante Schluckstörung bei 15 (14,7 %) der 102 Patienten.

Der prätherapeutische BMI-Wert lag im Mittel bei 25,6 ± 4,9 kg/m^2^ (14,53–39,31 kg/m^2^). Gemäß Weltgesundheitsorganisation (WHO) [19] zeigten von den 102 Patienten 6 (5,8 %) ein Untergewicht (< 18,5 kg/m^2^), 43 (42,2 %) ein Normalgewicht (18,5–24,9 kg/m^2^), 31 (30,4 %) eine Präadipositas (25,0–29,9 kg/m^2^), 17 (16,4 %) eine Adipositas Grad I (30–34,9 kg/m^2^) und 5 (4,9 %) eine Adipositas Grad II (35,0–39,9 kg/m^2^).

Der ungewollte Gewichtsverlust betrug im Mittel 2,8 ± 4,5 kg (0–20 kg). Von den 102 Patienten wiesen 12 (11,8 %) einen ungewollten Gewichtsverlust ≤ 5 kg auf, 20 (19,6 %) > 5–10 kg, 5 (4,9 %) > 10–20 kg. Damit zeigten insgesamt 25 Patienten (24,5 %) einen Gewichtsverlust von über 5 kg. Bei 65 der 102 Patienten (63,7 %) blieb das Gewicht stabil.

Der gEAT-10-Gesamtscore variierte zwischen 0 und 40 und lag im Durchschnitt bei 8,2 ± 10,6. Die Werteverteilung von PAS, FOIS, VRS, BMI und gEAT-10, abhängig von möglichen Einflussfaktoren, ist Tab. 2 zu entnehmen.

**Table 2:** 

Einflussfaktoren	PAS	FOIS	VRS	BMI	gEAT-10
	*n* (%)	*n* (%)	*n* (%)	M + SD	M + SD
*Tumorstadium*					
I	0 (0,0)	0 (0,0)	0 (0,0)	28,6 ± 4,5	1,0 ± 1,7
II	0 (0,0)	0 (0,0)	0 (0,0)	24,9 ± 4,0	5,6 ± 8,6
III	1 (4,5)	0 (0,0)	2 (9,1)	26,6 ± 4,9	5,2 ± 6,7
IV	7 (11,3)	7 (11,3)	13 (21,0)	25,2 ± 5,1	10,4 ± 12,0
*Tumorlokalisation*					
Mundhöhle	1 (5,3)	0 (0,0)	1 (5,3)	27,7 ± 5,8	5,4 ± 8,2
Oropharynx	3 (6,7)	2 (4,4)	6 (13,3)	24,8 ± 4,0	8,6 ± 11,1
Hypopharynx/Larynx	4 (10,5)	5 (13,2)	8 (21,1)	25,7 ± 5,4	9,1 ± 11,2
*Alter (Jahre)*					
41–60	2 (4,9)	1 (2,4)	3 (7,3)	25,2 ± 5,3	6,4 ± 8,7
> 60	6 (9,8)	6 (9,8)	12 (19,7)	26,0 ± 4,7	9,4 ± 11,7
*Geschlecht*					
Weiblich	1 (8,3)	0 (0,0)	1 (5,6)	23,9 ± 4,9	9,4 ± 10,6
Männlich	7 (5,6)	7 (8,3)	14 (16,7)	26,0 ± 4,9	7,9 ± 10,7

*gEAT-10* German Eating Assessment Tool 10, *SD* Standardabweichung

Das Tumorstadium korrelierte schwach bis mittelstark mit allen drei Parametern: PAS (*ρ* = 0,284; *p* = 0,004), FOIS (*ρ* = −0,340; *p* < 0,001) und VRS (*ρ* = 0,350; *p* < 0,001), nicht jedoch mit dem BMI (*p* > 0,05).

Die Penetration/Aspiration, Oralisierungseinschränkung, Versorgungsrelevanz sowie BMI unterschieden sich nicht signifikant abhängig von der Tumorlokalisation (*p*s > 0,05), auch nicht bei den paarweisen Vergleichen einzelner Tumorlokalisationen, außer dass bei Patienten mit einem Larynx‑/Hypopharynxkarzinom auffälligere PAS-Werte erzielt wurden als bei solchen mit einem Mundhöhlenkarzinom (Z = −2,2; *p* = 0,029).

Der Zusammenhang zwischen Patientengeschlecht bzw. -alter und PAS, FOIS, VRS sowie BMI war nicht signifikant (*p*s > 0,05).

Es zeigten sich schwache, aber signifikante Spearman-Korrelationen zwischen BMI und PAS (*ρ* = −0,273; *p* = 0,006), FOIS (*ρ* = −0,257; *p* = 0,009) und VRS (*ρ* = −0,278; *p* = 0,005).

In den linearen Regressionen mit PAS, FOIS und VRS als abhängige Variablen wurde lediglich das Tumorstadium durchgehend als relevanter Einflussfaktor detektiert (Tab. 3): je höher das Tumorstadium, desto höher die Skalenwerte. Niedrigere BMI-Werte waren mit dem weiblichen Geschlecht und auffälligeren Werten in der Versorgungsrelevanz assoziiert.

**Table 3:** 

	PAS	FOIS	VRS	BMI
*Gesamtmodell: F*	3,99^**^	3,97^**^	4,88^**^	4,42^**^
*Korrigiertes Nagelkerkes R*^*2*^	0,11	0,11	0,13	0,15
*Tumorstadium: ϐ/T*	0,271/2,824^**^	0,298/3,096^**^	0,311/3,286^**^	−0,018/−0,185
*Tumorlokalisation: ϐ/T*	0,208/2,165^*^	0,122/1,269	0,188/1,982^*^	−0,081/−0,839
*Alter: ϐ/T*	0,104/1,083	0,140/1,463	0,132/1,394	0,142/1,497
*Geschlecht: ϐ/T*	−0,088/−0,915	−0,075/−0,778	−0,090/−0,956	−0,214/−2,278^*^
*VRS: ϐ/T*	–	–	–	−0,382/−3,784^***^
*Konstante: T*	−0,942	−1,004	−1,082	6,615^***^

^***^ *p* < 0,001; ^**^ *p* < 0,01; ^*^ *p* < 0,05

Auffälligere Befunde in gEAT-10 waren durchgehend signifikant mit auffälligeren PAS-, FOIS- und VRS-Skalenwerten sowie mit niedrigeren BMI-Werten assoziiert (Tab. 4). Die Stärke einzelner Korrelationen variierte zwischen minimal und mittelstark. Zusammenhänge zwischen gEAT-10 und PAS lagen ausnahmslos niedriger als solche mit VRS und vor allem FOIS.

**Table 4:** 

gEAT-10	PAS	FOIS	VRS	BMI
Item 1: Mein Schluckproblem führte zu Gewichtsverlust	0,315^**^	0,495^***^	0,419^***^	−0,489^***^
Item 2: Mein Schluckproblem beeinträchtigt meine Möglichkeit, zum Essen auszugehen	0,364^***^	0,628^***^	0,519^***^	−0,364^***^
Item 3: Das Schlucken von Flüssigkeit erfordert besondere Anstrengung	0,351^***^	0,390^***^	0,400^***^	−0,395^***^
Item 4: Das Schlucken von fester Nahrung erfordert besondere Anstrengung	0,419^***^	0,612^***^	0,574^***^	−0,384^***^
Item 5: Das Schlucken von Tabletten erfordert besondere Anstrengung	0,389^***^	0,585^***^	0,509^***^	−0,459^***^
Item 6: Schlucken ist schmerzhaft	0,149	0,369^***^	0,267^**^	−0,396^***^
Item 7: Die Freude am Essen ist durch mein Schlucken beeinträchtigt	0,384^***^	0,488^***^	0,454^***^	−0,334^***^
Item 8: Wenn ich schlucke, bleibt mir Nahrung im Hals stecken	0,376^***^	0,489^***^	0,464^***^	−0,359^***^
Item 9: Ich huste, wenn ich esse	0,218^*^	0,389^***^	0,326^***^	−0,327^***^
Item 10: Schlucken ist anstrengend	0,301^**^	0,538^***^	0,429^***^	−0,380^***^
*gEAT-10-Gesamtscore*	0,379^***^	0,582^***^	0,402^***^	−0,456^***^

*gEAT-10* German Eating Assessment Tool 10

^***^p < 0,001; ^**^ *p* < 0,01; ^*^ *p* < 0,05

Der tatsächliche Gewichtsverlust war bei Patienten mit auffälligeren Befunden im gEAT-10-Item 1 stärker ausgeprägt (*ρ* = 0,610; *p* < 0,001).

## Diskussion

Patienten mit einem Kopf-Hals-Tumor vor Beginn einer onkologischen Therapie zeigten ein seltenes Vorkommen einer objektivierbaren Schluckstörung in Bezug auf Penetration/Aspiration, Oralisierungseinschränkung und Versorgungsrelevanz. Wenn, so fand sich eine Schluckstörung v. a. bei Patienten mit UICC-Stadien III und IV. Damit zeigte sich bereits prätherapeutisch eine Tendenz des objektiv ermittelten Schluckvermögens, die in früheren Studien bei Patienten nach onkologischer Therapie nachgewiesen wurde: je höher das Tumorstadium, desto ausgeprägter die Schluckstörung [20–22].

Unterschiede im Schweregrad einer Schluckstörung je nach Tumorlokalisation waren minimal ausgeprägt. Patienten mit einem Larynx- bzw. Hypopharynx-Karzinom zeigten etwas häufiger Auffälligkeiten hinsichtlich Aspiration/Penetration und einer therapierelevanten Schluckstörung als Patienten mit einem Mundhöhlenkarzinom, was ob der Lokalisation einen zu erwartenden Befund darstellt [23]. Patientenalter und -geschlecht waren dagegen mit allen drei Parametern (PAS, FOIS, VRS) nicht signifikant assoziiert.

Die Patienten beurteilten ihr Schluckvermögen subjektiv mit einem durchschnittlichen gEAT-10-Gesamtscore von 8 als auffällig, legt man das von Belafsky et al. [11] definierte Cut-off-Kriterium ≥ 3 zugrunde. Es zeigte sich dabei eine hohe Übereinstimmung der objektivierbaren Schluckbeeinträchtigung mit der subjektiven Einschätzung der Patienten, die bereits in einigen früheren Studien für die prätherapeutische Phase demonstriert wurde [24, 25].

Der Ernährungszustand war mit einem durchschnittlichen BMI von 26 kg/m^2^ gemäß WHO-Einteilung eher als präadipös zu werten. Nur knapp 6 % aller Patienten wiesen eine Malnutrition auf, 25 % einen ungewollten Gewichtsverlust oberhalb von 5 kg, was trotz des klaren Überwiegens eines normalgewichtigen oder sogar adipösen Ernährungszustands einen Hinweis möglicher Malnutritionsgefährdung bildet [26]. Dieses Ergebnis sollte daher ob der Tragweite hinsichtlich der Beeinflussung des onkologischen Therapieoutcomes [27] mit geeigneten Erfassungstools prospektiv untersucht werden.

Während sich der BMI als unabhängig von Tumorlokalisation, -stadium und Patientenalter erwies, bestand ein signifikanter Zusammenhang zum einen zur Schwere einer Schluckstörung, gemessen an der Versorgungsrelevanz, zum anderen zum Patientengeschlecht: Höhere BMI-Werte fanden sich v. a. bei Patienten mit keiner oder nur geringer Beeinträchtigung des Schluckens und beim männlichen Geschlecht.

Die hier mittels Regressionsanalysen untersuchten Faktoren hinsichtlich einer möglichen Einflussnahme auf Penetration/Aspiration, Oralisierungseinschränkung, Versorgungsrelevanz und BMI ergaben eine Varianzerklärung von maximal 15 %, was bedeutet, dass weitere wichtige Einflussfaktoren existieren, die hier nicht berücksichtigt wurden.

Vergleicht man die hier untersuchte Patientenpopulation mit den 2019 vom Robert Koch-Institut (RKI) publizierten Zahlen zu Patienten mit einem Kopf-Hals-Tumor [28], so ist die hier verwendete Stichprobe repräsentativ in Bezug auf Tumorlokalisation und Patientenalter, nicht jedoch in Bezug auf das Geschlechtsverhältnis. Hier sind die Männer in Relation zu den RKI-Daten überrepräsentiert, was die Vergleichbarkeit zwischen Männern und Frauen in der aktuellen Studie zusätzlich reduziert. Da generell immer deutlich mehr Männer von einer Kopf-Hals-Tumorerkrankung betroffen sind, sind per se diskrepante Ergebnisse zwischen dieser Art von uni- und multivariaten Analysen zu erwarten. Um eine Generalisierung der vorliegenden Studienergebnisse vornehmen zu können, bedarf es daher einer Vergrößerung der Studienpopulation. Eine größere Fallzahl ließe dann auch die separierte Analyse des Schluckvermögens für die unterschiedlichen Tumorlokalisationen (z. B. Hypopharynx/Larynx) zu.

Nichtsdestotrotz zeigen die hier präsentierten prospektiv erhobenen Studienergebnisse, dass Kopf-Hals-Tumor-Patienten bereits prätherapeutisch sowohl objektiv als auch subjektiv Schluckstörungen ebenso wie Auffälligkeiten des Ernährungszustands aufweisen können. Diese gilt es, in der weiteren onkologischen Behandlungsplanung bzw. im Verlauf einer onkologischen Therapie zu berücksichtigen, um weitere Folgekomplikationen zu verhindern bzw. den Patienten trotz aller Einschränkungen das Maximum an Lebensqualität zu ermöglichen.

### Infobox 1. Die 8-Punkte-Penetrations-Aspirations-Skala nach Rosenbek [15]

- 1 Material dringt nicht in den Luftweg ein
- 2 Material dringt in den Luftweg ein, verbleibt oberhalb der Stimmlippen und wird aus dem Luftweg ausgestoßen
- 3 Material dringt in den Luftweg ein, verbleibt oberhalb der Stimmlippen und wird nicht aus dem Luftweg ausgestoßen
- 4 Material dringt in den Luftweg ein, kontaktiert die Stimmlippen und wird aus dem Luftweg ausgestoßen
- 5 Material dringt in den Luftweg ein, kontaktiert die Stimmlippen und wird nicht aus dem Luftweg ausgestoßen
- 6 Material dringt in den Luftweg ein, passiert bis unter die Stimmlippen und wird in den Larynx hinein oder aus dem Luftweg ausgestoßen
- 7 Material dringt in den Luftweg ein, passiert bis unter die Stimmlippen und wird nicht aus der Trachea ausgestoßen, trotz Bemühung
- 8 Material dringt in den Luftweg ein, passiert bis unter die Stimmlippen, und es wird keine Bemühung zum Ausstoßen unternommen

### Infobox 2. Die Functional Oral Intake Scale nach Crary [17] (invertiert)

- 1 Vollständig orale Diät ohne Einschränkungen
- 2 Vollständig orale Diät mit verschiedenen Konsistenzen, ohne spezielle Zubereitung, aber mit Einschränkungen spezifischer Lebensmittel
- 3 Vollständig orale Diät mit verschiedenen Konsistenzen, aber spezieller Zubereitung oder Kompensation
- 4 Vollständig orale Diät mit einer Konsistenz
- 5 Sondenabhängig mit konsistenten Versuchen oraler Aufnahme von Nahrung oder Flüssigkeiten
- 6 Sondenabhängig mit minimalen Versuchen oraler Gabe von Nahrung oder Flüssigkeiten
- 7 Nichts über den Mund

## Fazit für die Praxis

- Trotz hier niedriger Prävalenzrate einer versorgungsrelevanten Schluckstörung und eines Untergewichts bestehen Hinweise darauf, dass v. a. Patienten mit höheren Tumorstadien und Larynx‑/Hypopharynx-Karzinomen bereits prätherapeutisch bzgl. ihres Schluckvermögens und Nutritionsstatus beeinträchtigt sind.
- Auch wenn nur sehr wenige Patienten bei Diagnosestellung einen BMI unter der Norm aufweisen, berichten 25 % über einen ungewollten Gewichtsverlust von > 5 kg.
- Daher sollten alle Kopf-Hals-Tumor-Patienten v. a. höheren Tumorstadiums hinsichtlich Schluck- und Nutritionsstatus systematisch bereits vor Beginn der onkologischen Therapie untersucht werden.
- Das Ziel: eine personenzentrierte Optimierung der peritherapeutischen onkologischen Versorgung von Kopf-Hals-Tumor-Patienten.
